# Social determinants of health predict readmission following COVID-19 hospitalization: a health information exchange-based retrospective cohort study

**DOI:** 10.3389/fpubh.2024.1352240

**Published:** 2024-03-27

**Authors:** Micaela N. Sandoval, Jennifer L. Mikhail, Melyssa K. Fink, Guillermo A. Tortolero, Tru Cao, Ryan Ramphul, Junaid Husain, Eric Boerwinkle

**Affiliations:** ^1^Department of Epidemiology, Human Genetics, and Environmental Sciences, University of Texas Health Science Center at Houston School of Public Health, Houston, TX, United States; ^2^Greater Houston HealthConnect, Houston, TX, United States; ^3^Department of Biostatistics and Data Science, University of Texas Health Science Center at Houston School of Public Health, Houston, TX, United States

**Keywords:** COVID-19, social determinants of health, epidemiology, clinical outcomes, infectious disease, healthcare utilization, health disparities, hospitalization

## Abstract

**Introduction:**

Since February 2020, over 104 million people in the United States have been diagnosed with SARS-CoV-2 infection, or COVID-19, with over 8.5 million reported in the state of Texas. This study analyzed social determinants of health as predictors for readmission among COVID-19 patients in Southeast Texas, United States.

**Methods:**

A retrospective cohort study was conducted investigating demographic and clinical risk factors for 30, 60, and 90-day readmission outcomes among adult patients with a COVID-19-associated inpatient hospitalization encounter within a regional health information exchange between February 1, 2020, to December 1, 2022.

**Results and discussion:**

In this cohort of 91,007 adult patients with a COVID-19-associated hospitalization, over 21% were readmitted to the hospital within 90  days (*n* = 19,679), and 13% were readmitted within 30  days (*n* = 11,912). In logistic regression analyses, Hispanic and non-Hispanic Asian patients were less likely to be readmitted within 90  days (adjusted odds ratio [aOR]: 0.8, 95% confidence interval [CI]: 0.7–0.9, and aOR: 0.8, 95% CI: 0.8–0.8), while non-Hispanic Black patients were more likely to be readmitted (aOR: 1.1, 95% CI: 1.0–1.1, *p* = 0.002), compared to non-Hispanic White patients. Area deprivation index displayed a clear dose–response relationship to readmission: patients living in the most disadvantaged neighborhoods were more likely to be readmitted within 30 (aOR: 1.1, 95% CI: 1.0–1.2), 60 (aOR: 1.1, 95% CI: 1.2–1.2), and 90  days (aOR: 1.2, 95% CI: 1.1–1.2), compared to patients from the least disadvantaged neighborhoods. Our findings demonstrate the lasting impact of COVID-19, especially among members of marginalized communities, and the increasing burden of COVID-19 morbidity on the healthcare system.

## 1 Introduction

Since February 2020, over 104 million people in the United States have been diagnosed with SARS-CoV-2 infection, or COVID-19, with over 8.5 million, or 28,812 per hundred thousand population, reported in the state of Texas (1). The risk of SARS-CoV-2 exposure, progression to clinical disease, and severe outcomes such as hospitalization and death depend on both individual and societal factors (2–6). However, there is increasing recognition of significant rates of severe COVID-19 outcomes and post-acute syndromes, including long COVID, among populations previously thought to be ‘low-risk’ (7–9). Furthermore, the absolute risk of outcomes, such as hospitalization and death, have changed over time as novel SARS-CoV-2 variants emerged, vaccinations increased, and public health policies adapted to local epidemic dynamics (10–12).

As the pandemic progressed, social determinants of health, including demographic, financial, and social factors, emerged as significant contributors to adverse COVID-19 outcomes. Investigations have highlighted the risk of SARS-CoV-2 exposure among low-income, minority, and immigrant populations (13), and the impracticality of quarantine and isolation guidelines in high-density housing and other communal settings (14). Additionally, disparities in healthcare utilization among members of different socioeconomic groups were well documented before and during the COVID-19 pandemic (15–17). Hospital visits, especially unplanned readmissions, are important metrics not only of patient health, but also of healthcare practices, population health, and care costs (18, 19).

While increasing age and comorbidity burden have been identified as independent risk factors for COVID-19-related hospitalization and readmission, the relationship between readmission across healthcare systems and social determinants of health in the United States has been described in only a few studies (4, 20, 21). Therefore, the current study aimed to investigate 30, 60, and 90-day readmission outcomes among patients with a COVID-19-associated inpatient hospitalization encounter identified within a regional health information exchange between February 1, 2020, to December 1, 2022.

## 2 Methods

### 2.1 Health information exchange

Greater Houston Healthconnect (GHH) is the regional health information exchange (HIE) for Southeast Texas. GHH collects prospective and retrospective health data from approximately 15.5 million unique patients from more than 75 Texas counties and 40 Louisiana parishes through partnerships with more than 150 member hospitals, over 2,000 ambulatory practices, and several local public health departments. In practice, HL7 version 2 real-time feeds and Consolidated Continuity of Care Documents (C-CDA) are converted to a relational database with individual patients’ longitudinal electronic health data. While the primary objective of any HIE is to facilitate clinical care by supporting the efficient exchange of clinical information, these large EHR datasets are increasingly being utilized for treatment, payment, and operations-related research (22, 23).

### 2.2 Identification of COVID-19 cases

COVID-19 cases were defined as any patient with either: A COVID-19 diagnosis identified through ICD-10 or SNOMED CT codes (see Supplementary 1 for the codeset); A positive diagnostic laboratory test for SARS-CoV-2, including nucleic acid amplification tests and antigen tests (antibody tests were excluded); And a case report documented by local public health departments. Patients for whom a COVID-19 identification date could not be determined were excluded.

### 2.3 Study population

The study area for this investigation covered most of Southeast Texas and included Brazoria, Burleson, Chambers, Fort Bend, Galveston, Grimes, Hardin, Harris, Jasper, Jefferson, Liberty, Madison, Matagorda, Montgomery, Nueces, Orange, Polk, San Jacinto, San Patricio, Walker, Waller, and Wharton counties, where a high proportion of hospitals are GHH members. Patients’ residential addresses were extracted at the time of the initial data pull (December 2022). Patients with an ‘inpatient’ encounter beginning within 7 days (+/−) of any COVID-19 identification date who resided within the study area were eligible for inclusion in the COVID-19 inpatient cohort. ‘Emergency Room’ type encounters were not included in the inpatient cohort.

### 2.4 Exclusion criteria

Pediatric patients (<18 years of age), patients who were pregnant or delivering at their index encounter, patients who expired during their index encounter, and patients residing outside of the study area were excluded from readmission analyses. Pregnant patients were excluded from readmission analyses due to the likelihood of subsequent hospital encounters unrelated to COVID-19, i.e., labor and delivery encounters.

### 2.5 Study outcomes

The primary outcome was all-cause readmission, defined as any subsequent inpatient hospital encounter beginning within 90 days from discharge from the index encounter. Patients for whom readmission status could not be determined (e.g., a post-discharge encounter that was not clearly a readmission) were excluded from this analysis.

### 2.6 Study exposures

Patient demographics, including age at index encounter admission, sex, race, and ethnicity, were extracted directly from the EHR. The Charlson Comorbidity Index (CCI) was calculated as a measure of overall comorbidity burden (24, 25); individual CCI components were extracted by searching ICD-10-CM (26) and SNOMED CT (27) diagnosis codes associated with the index encounter as well as up to 3 years prior to the index encounter. Peaks in Texas COVID-19 incidence were used to categorize COVID-19 admissions to further reflect local epidemic dynamics (28).

### 2.7 Geographic information

COVID-19 patient hospitalization data were collected for the state of Texas from publicly available Department of State Health Services (DSHS) datasets.^1^ Publicly available geographic information system (GIS) datasets were collected from the Texas Parks and Wildlife Department, the Texas Department of Transportation, the US Census repository, and DSHS. Ecological measures of socioeconomic disadvantage, including the Area Deprivation Index (ADI), which measures relative deprivation between all census block groups by state (29, 30) and the Social Vulnerability Index (SVI), which measures relative vulnerability to disaster among all census tracts in the state (31) were calculated from the geocoded patient-provided home addresses collated and analyzed December 2022. Heat maps were created by calculating kernel density estimates from patients’ residential addresses; low-density values (<15th quantile) were truncated to preserve patient privacy. All geospatial analyses were performed on ArcGIS Pro version 3.1.1 (ESRI, Redlands, CA).

### 2.8 Statistical analyses

Demographic and clinical data were reported as frequencies and proportions for categorical variables and as the median and interquartile range (IQR) for continuous variables. Logistic regression modeling was performed to identify risk factors for readmission at 30, 60, and 90 days from discharge; crude and adjusted odds ratios and 95% confidence intervals are provided as estimates of risk for each outcome. Variable selection for the multivariable models was based on *a priori* clinical importance. For survival analyses, time zero was the date of discharge from the index encounter, event time was the date of first readmission, and data were censored at 90 days. All analyses were performed on Stata MP version 17.0 (StataCorp LLC, College Station, TX, United States). A *p*-value of <0.05 was considered nominally significant; a conservative, Bonferroni-corrected statistical significance threshold of 0.00625 was utilized in model-building.

### 2.9 Ethics statement

This retrospective registry-based study was approved by the Western Institutional Review Board as a quality improvement study and granted a waiver of informed consent (#1–1,053,411-1).

## 3 Results

### 3.1 Study population

**Figure 1:**
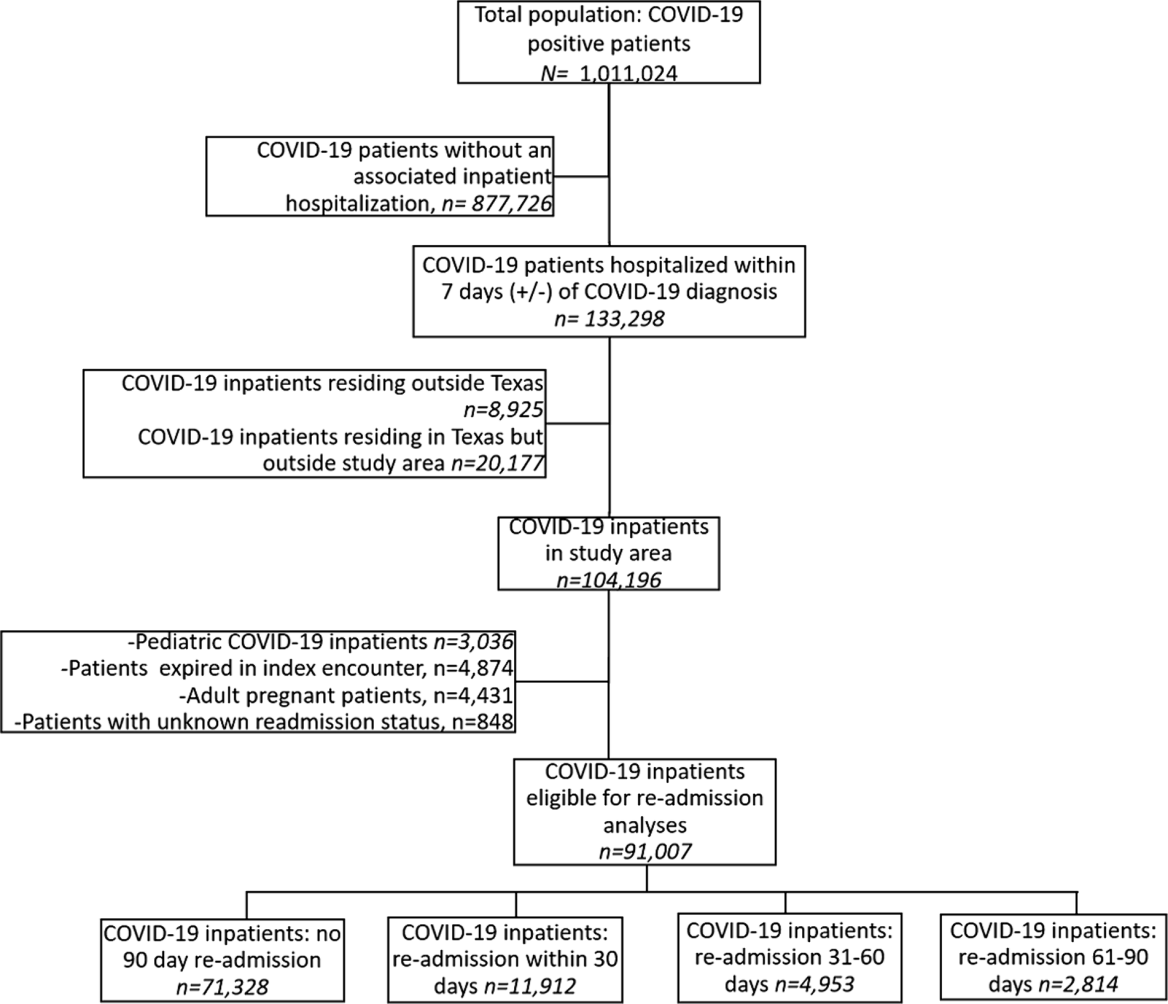
Study flowchart.

**Figure 2:**
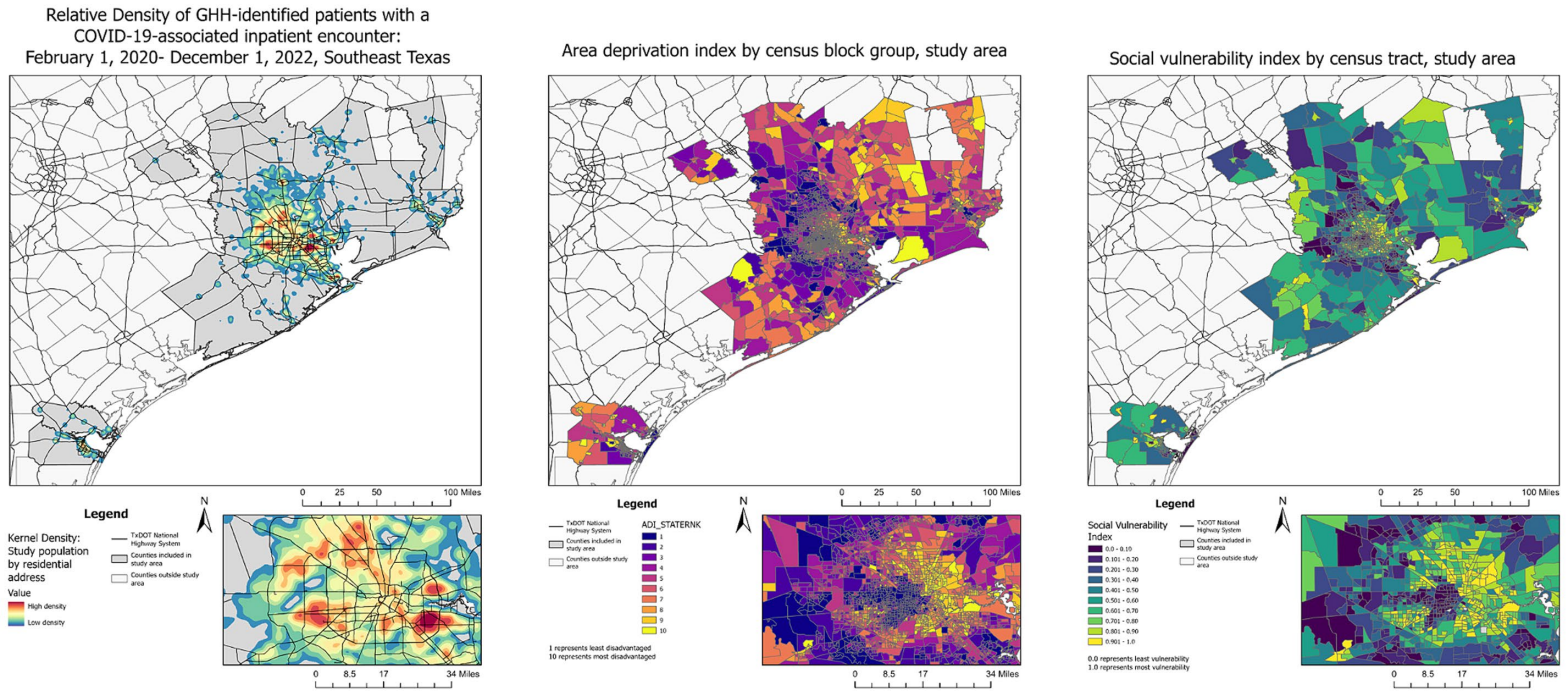
Relative density of GHH-identified patients with a COVID-19-associated inpatient encounter: February 1, 2020–December 1, 2022.

**Figure 3:**
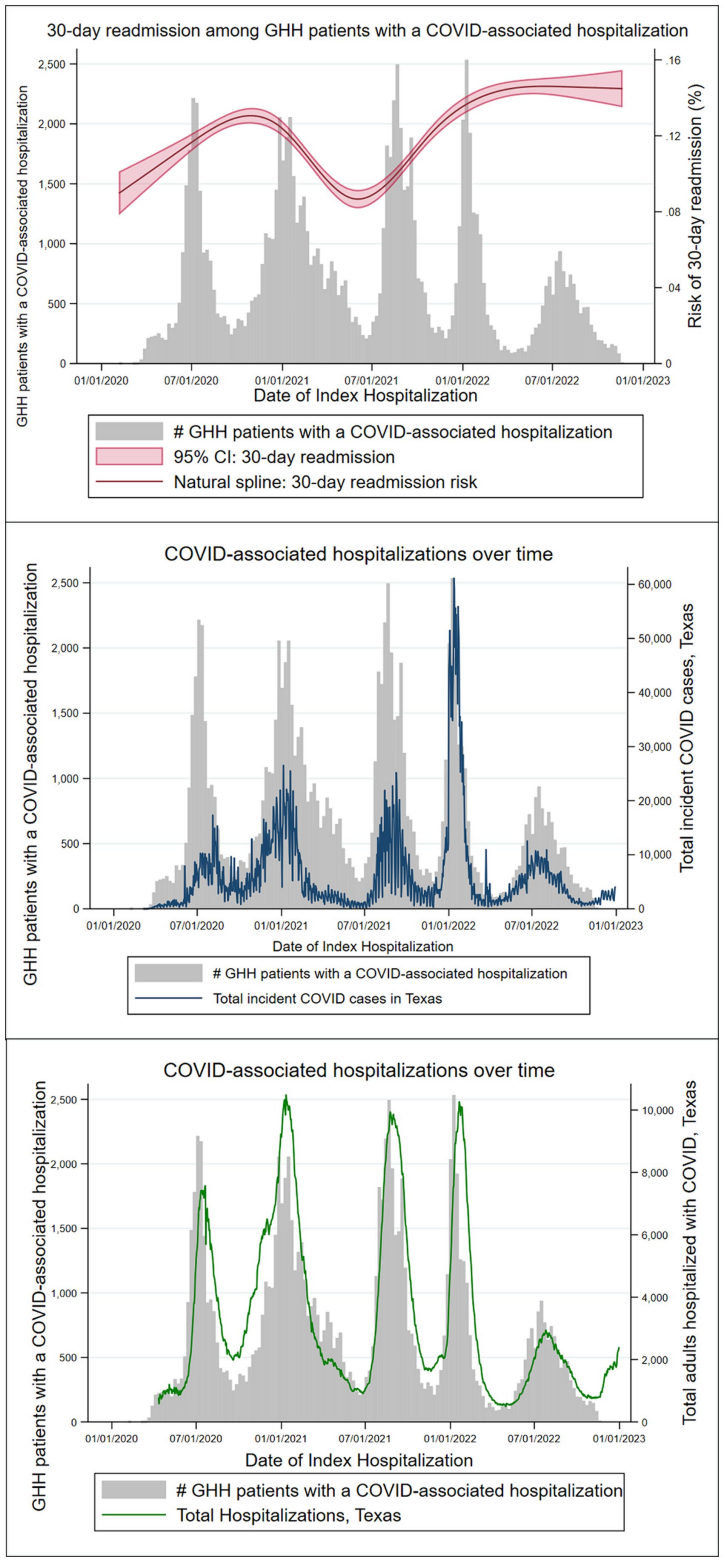
GHH patients with a COVID-19-associated hospitalization.

From February 1, 2020, to December 1, 2022, 1,011,024 patients were identified as COVID-19 cases by diagnosis, laboratory testing, or local public health case reporting, of whom 133,298 (13%) had an inpatient hospital encounter within 7 days (+/−) of a COVID-19 identification date (Figure 1). Of these, 104,196 had a residential address within the study area, making-up the COVID-19 inpatient cohort (Figure 2). Of the inpatients, 80,253 were identified as COVID-19 cases during their index hospitalization. In this COVID-19 inpatient cohort, trends in inpatient admissions mirrored total COVID-19 incidence and total COVID-19 hospitalizations for the state of Texas (Figure 3). The median age at admission was 57.4 (IQR: 40.4–71.0), and 51,062 (49%) scored zero on the CCI (Table 1).

**Table 1:** Characteristics of GHH patients with a COVID-19-associated inpatient hospitalization.

	Total
Characteristics	*N* = 104,196
Demographics	*n (%)*
Age (years)	
under 18	3,036 (2.9%)
18–29	9,945 (9.5%)
30–49	26,703 (25.6%)
50–69	36,521 (35.1%)
70+	27,991 (26.9%)
Sex	
Male	49,492 (47.5%)
Female, non-pregnant	50,027 (48.0%)
Pregnant female	4,534 (4.4%)
Unknown	143 (0.1%)
Race/Ethnicity	
Non-Hispanic White	46,071 (44.2%)
Non-Hispanic Black	19,664 (18.9%)
Non-Hispanic Asian	2,927 (2.8%)
Non-Hispanic American Indian/Alaska Native	371 (0.4%)
Non-Hispanic Native Hawaiian/ Pacific Islander	190 (0.2%)
Non-Hispanic Other	6,620 (6.4%)
Hispanic	27,040 (26.0%)
Missing	1,313 (1.1%)
Social vulnerability index	
1 (Least Vulnerable)	7,102 (6.8%)
2	8,327 (8.0%)
3	8,469 (8.1%)
4	10,364 (10.0%)
5	10,645 (10.2%)
6	9,513 (9.1%)
7	12,025 (11.5%)
8	13,057 (12.5%)
9	13,464 (12.9%)
10 (Most vulnerable)	11,146 (10.7%)
Missing	84 (0.1%)
Area deprivation index	
1 (Least disadvantaged)	7,523 (7.2%)
2	9,638 (9.2%)
3	10,499 (10.1%)
4	11,917 (11.4%)
5	11,690 (11.2%)
6	13,436 (12.9%)
7	12,600 (12.1%)
8	10,774 (10.3%)
9	9,450 (9.1%)
10 (Most disadvantaged)	5,952 (5.7%)
Missing	717 (0.7%)
Charlson Comorbidity Index, median (IQR)	1 (0–2)
Chronic pulmonary disease	11,936 (11.5%)
Cerebrovascular disease	3,729 (3.6%)
Dementia	4,255 (4.1%)
Diabetes without complications	22,160 (21.3%)
Diabetes with complications	5,239 (5.0%)
Congestive heart failure	10,111 (9.7%)
Hemiplegia	971 (0.9%)
Myocardial infarction history	4,899 (4.7%)
Mild liver disease	3,095 (3.0%)
Moderate to severe liver disease	805 (0.8%)
Mild to moderate renal disease	7,134 (6.8%)
Severe renal disease	4,332 (4.2%)
Peptic ulcer disease	608 (0.6%)
Peripheral vascular disease	3,011 (2.9%)
Rheumatic disease	1,387 (1.3%)
HIV infection	555 (0.5%)
HIV infection with complications	471 (0.5%)
Malignant neoplasm	5,072 (4.9%)
Solid tumor	849 (0.8%)
Date of index hospitalization	
February 1, 2020- September 15, 2020	18,347 (17.6%)
September 16, 2020- June 20, 2021	34,919 (33.5%)
June 21, 2021- November 20, 2021	23,319 (22.4%)
November 21, 2021- April 15, 2022	15,267 (14.6%)
April 16, 2022- November 30, 2022	12,344 (11.9%)
Length of stay (days), median (IQR)	4 (2–9)
Financial class (index hospitalization)	
Private insurance	21,729 (20.9%)
Medicare/Medicaid alone	25,542 (24.5%)
Medicare/Medicaid plus private insurance	23,533 (22.6%)
Self-Pay/Safety net	2,041 (2.0%)
Military or government	843 (0.8%)
COVID pay	1,479 (1.4%)
Other	436 (0.4%)
Unknown*	28,593 (27.4%)
Discharge disposition	
Home	47,318 (45.4%)
Transfer (facility)	653 (0.6%)
Expired	4,875 (4.7%)
Transfer (SNF/Nursing home)	3,467 (3.3%)
Transfer (Rehab/LTAC)	2,573 (2.5%)
Against medical advice	1,025 (1.0%)
Hospice	1,376 (1.3%)
Still patient	743 (0.7%)
Other	1,425 (1.4%)
Unknown	40,741 (39.1%)

*Unknown indicates insurance information was not reported, includes uninsured patients.NH, Non-Hispanic; IQR, Interquartile range; SNF, Skilled nursing facility; LTAC, long-term acute care.

At their index hospital encounter, 21% of patients were privately insured, 47% were Medicare or Medicaid clients, and 27% had no payer information available (Table 1). Index inpatient encounters that noted the death of the patient occurred 4,875 (5%) times and were excluded from these readmission analyses. In total, 91,007 adult inpatients were included in these readmission analyses, of whom 11,912 (13%) were readmitted within 30 days of discharge from their index encounter (Figure 1). Additionally, 14,479 (74%) of readmitted patients returned to the same hospital, while 5,200 (26%) were admitted to a different hospital from their index encounter. Of the 19,679 patients who were readmitted within 90 days, 822 (4%) expired during their first readmission encounter. Diagnoses associated with index and readmission encounters are shown in Supplementary 2.

### 3.2 Readmission analyses

**Figure 4:**
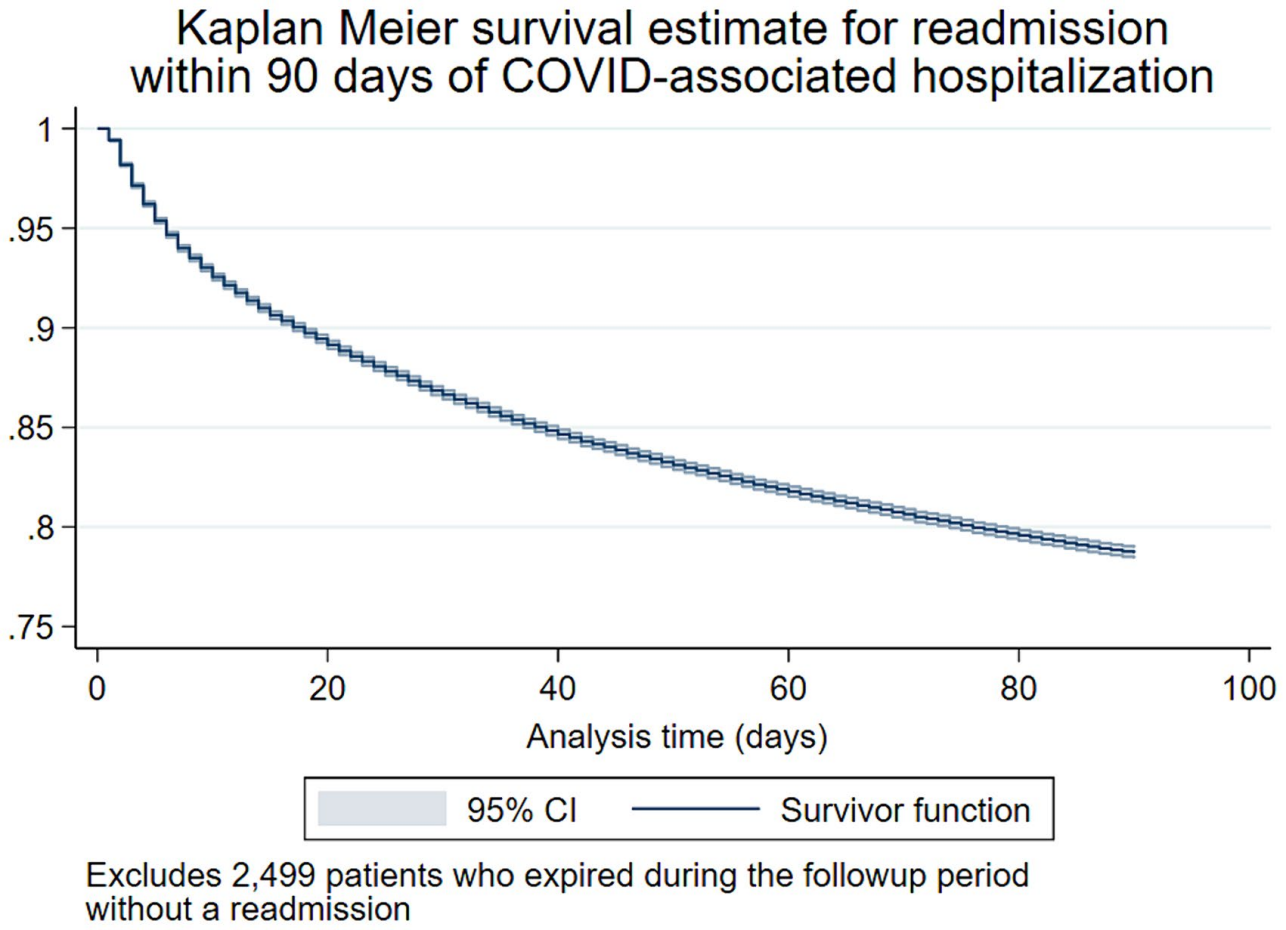
Kaplan–Meier survival estimate for 90-day readmission following COVID-19-associated hospitalization.

**Figure 5:**
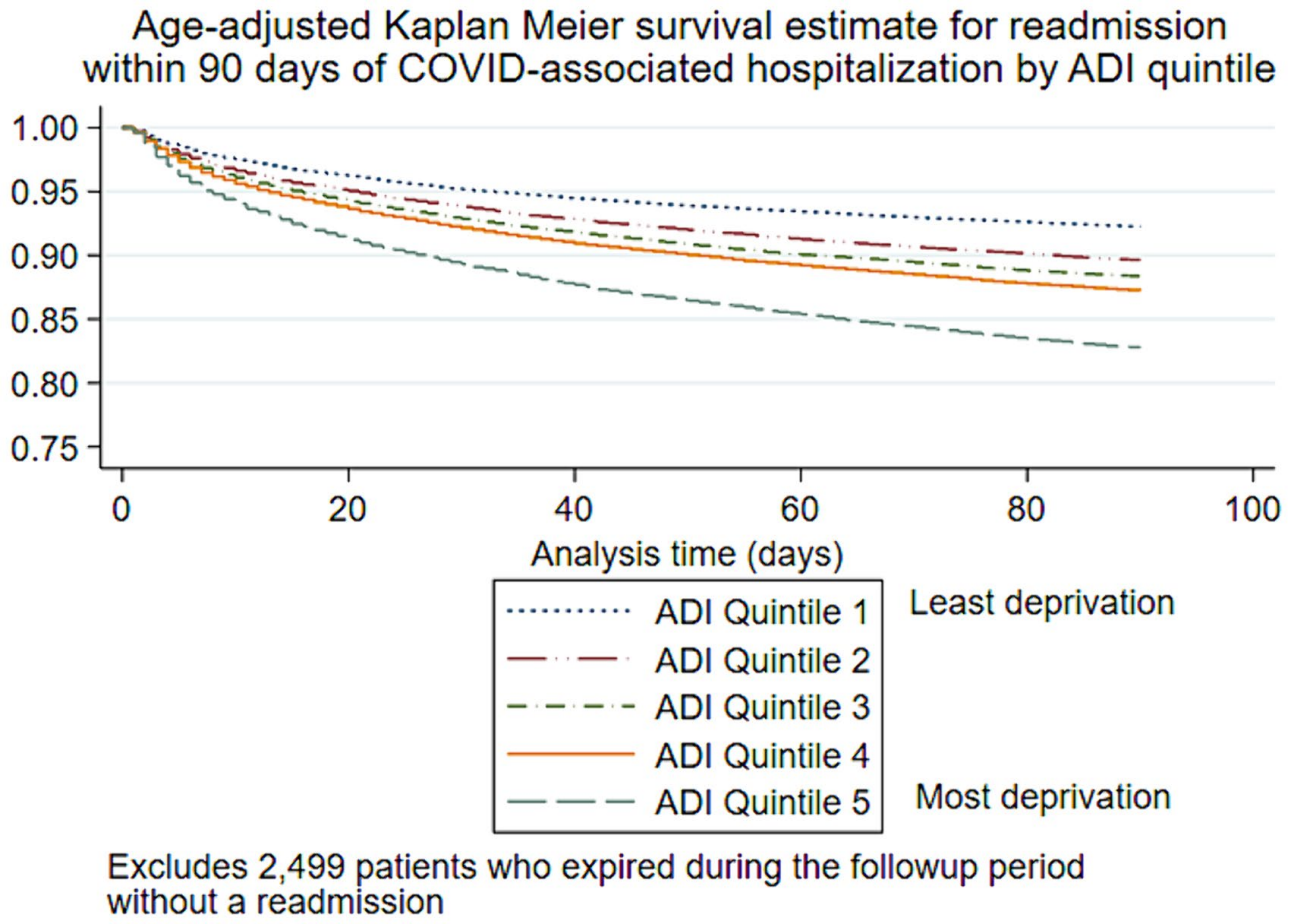
Age-adjusted Kaplan–Meier survival estimate for 90-day readmission following COVID-19-associated hospitalization by area deprivation index.

Univariable logistic regression analyses for 30, 60, and 90-day readmission are shown in Table 2, and Kaplan–Meier survival curves for time to readmission are shown in Figures 4 5. Patients who expired during the observation period without a readmission (*n* = 2,499 patients) were excluded from Kaplan–Meier analyses. In multivariable logistic regression analysis, increasing age at encounter was significantly associated with 30, 60, and 90-day readmission (Table 3). Hispanic and non-Hispanic Asian patients were less likely to be readmitted within 90 days (aOR: 0.8, 95% CI: 0.7–0.9, and aOR: 0.8, 95% CI: 0.8–0.8), while non-Hispanic Black patients were more likely to be readmitted (aOR: 1.1, 95% CI: 1.0–1.1, *p* = 0.002), compared to non-Hispanic White patients. Living in neighborhoods with higher relative disadvantage was a significant risk factor in 30, 60, and 90-day readmission models. Increasing CCI scores were a risk factor in all readmission models. Medicare/Medicaid clients and patients without a named payor were more likely to be readmitted compared to patients with commercial insurance (aOR: 1.4, 95% CI: 1.3–1.5, and aOR: 1.3, 95% CI: 1.2–1.3), while patients with index encounters primarily covered by special COVID-19 funds were less likely to be readmitted within 90 days (aOR: 0.7, 95% CI: 0.5–0.8). Length of stay <2 days or ≥ 10 days were both risk factors for 90-day readmission compared to stays 4 to 5 days long (aOR: 2.0, 95% CI: 1.9–2.1, and aOR: 1.1, 95% CI: 1.0–1.1). To address the problem of competing risks of mortality and readmission and identify possible survivorship biases, we conducted additional analyses of 30-day mortality and readmission as a composite outcome (Supplementary 3). Receiver operating characteristic curves are displayed in Supplementary 4; area under the curve for each multivariable regression model are presented in the table legend (Figure 5).

**Table 2:** Univariate logistic regression: readmission among patients with a COVID-19-associated inpatient hospitalization.

Univariate logistic regression, *N* = 91,007	30 day readmission		60 day readmission		90 day readmission	
Events	*n* = 11,912		*n* = 16,865		*n* = 19,679	
	*OR (95% CI)*	*p value*	*OR (95% CI)*	*p value*	*OR (95% CI)*	*p value*
Age (years)						
18–29	REF		REF		REF	
30–49	0.99 (0.91–1.08)	0.874	0.97 (0.90–1.05)	0.472	0.97 (0.91–1.04)	0.405
50–69	1.33 (1.23–1.44)	<0.001	1.35 (1.26–1.45)	<0.001	1.33 (1.25–1.42)	<0.001
70+	1.69 (1.55–1.83)	<0.001	1.80 (1.68–1.94)	<0.001	1.82 (1.70–1.94)	<0.001
Sex						
Male	REF		REF		REF	
Female, non-pregnant	1.02 (1.98–1.06)	0.358	1.06 (1.03–1.10)	0.001	1.09 (1.05–1.12)	<0.001
Race/Ethnicity						
Non-Hispanic White	REF		REF		REF	
Non-Hispanic Black	1.06 (1.00–1.11)	0.034	1.08 (1.04–1.13)	<0.001	1.08 (1.04–1.12)	<0.001
Non-Hispanic Asian	0.83 (0.74–0.94)	0.004	0.77 (0.69–0.86)	<0.001	0.73 (0.66–0.81)	<0.001
Non-Hispanic American Indian/Alaska Native	0.94 (0.68–1.29)	0.702	0.83 (0.62–1.10)	0.2	0.86 (0.66–1.12)	0.263
Non-Hispanic Native Hawaiian/ Pacific Islander	1.07 (0.69–1.65)	0.761	1.06 (0.73–1.55)	0.752	0.97 (0.67–1.40)	0.868
Non-Hispanic Other	0.75 (0.69–0.82)	<0.001	0.70 (0.65–0.76)	<0.001	0.69 (0.64–0.75)	<0.001
Hispanic	0.77 (0.73–0.81)	<0.001	0.72 (0.69–0.75)	<0.001	0.70 (0.67–0.73)	<0.001
Missing	0.15 (0.08–0.30)	<0.001	0.12 (0.07–0.22)	<0.001	0.14 (0.08–0.23)	<0.001
Social vulnerability index						
Quintile 1 (Least Vulnerable)	REF		REF		REF	
Quintile 2	1.07 (1.00–1.14)	0.063	1.07 (1.01–1.14)	0.024	1.08 (1.02–1.15)	0.005
Quintile 3	1.10 (1.03–1.18)	0.006	1.08 (1.02–1.14)	0.01	1.07 (1.01–1.13)	0.014
Quintile 4	1.23 (1.16–1.32)	<0.001	1.21 (1.14–1.28)	<0.001	1.22 (1.16–1.29)	<0.001
Quintile 5 (Most Vulnerable)	1.17 (1.10–1.25)	<0.001	1.14 (1.08–1.20)	<0.001	1.15 (1.09–1.21)	<0.001
Area deprivation index						
Quintile 1 (Least Disadvantaged)	REF		REF		REF	
Quintile 2	1.01 (0.95–1.08)	0.737	1.06 (1.00–1.12)	0.048	1.07 (1.02–1.13)	0.008
Quintile 3	1.10 (1.03–1.17)	0.004	1.13 (1.07–1.19)	<0.001	1.13 (1.07–1.19)	<0.001
Quintile 4	1.15 (1.08–1.22)	<0.001	1.17 (1.10–1.23)	<0.001	1.19 (1.13–1.25)	<0.001
Quintile 5 (Most Disadvantaged)	1.21 (1.13–1.30)	<0.001	1.23 (1.16–1.31)	<0.001	1.26 (1.19–1.33)	<0.001
Charlson Comorbidity Index	1.20 (1.19–1.21)	<0.001	1.24 (1.23–1.25)	<0.001	1.26 (1.25–1.27)	<0.001
Date of index hospitalization						
February 1, 2020-September 15, 2020	REF		REF		REF	
September 16, 2020- June 20, 2021	0.99 (0.94–1.05)	0.757	0.99 (0.94–1.04)	0.709	0.98 (0.93–1.02)	0.319
June 21, 2021- November 20, 2021	0.82 (0.77–0.87)	<0.001	0.82 (0.77–0.86)	<0.001	0.80 (0.76–0.84)	<0.001
November 21, 2021- April 15, 2022	1.37 (1.28–1.46)	<0.001	1.49 (1.40–1.57)	<0.001	1.54 (1.46–1.63)	<0.001
April 16, 2022- November 30, 2022	1.32 (1.23–1.42)	<0.001	1.48 (1.40–1.58)	<0.001	1.43 (1.35–1.52)	<0.001
Length of stay (index hospitalization)						
<2 days	2.05 (1.93–2.18)	<0.001	1.86 (1.76–1.96)	<0.001	1.74 (1.66–1.83)	<0.001
2–3 days	1.18 (1.11–1.26)	<0.001	1.10 (1.04–1.16)	0.001	1.07 (1.02–1.13)	0.007
4–5 days	REF		REF		REF	
6–7 days	1.03 (0.95–1.11)	0.487	1.07 (1.00–1.14)	0.039	1.08 (1.01–1.14)	0.016
8–9 days	1.18 (1.08–1.29)	<0.001	1.19 (1.10–1.28)	<0.001	1.16 (1.08–1.25)	<0.001
10+ days	1.23 (1.15–1.31)	<0.001	1.32 (1.25–1.39)	<0.001	1.28 (1.22–1.35)	<0.001
Financial class (index hospitalization)						
Private insurance	REF		REF		REF	
Medicare/Medicaid alone	1.61 (1.52–1.71)	<0.001	1.70 (1.62–1.79)	<0.001	1.76 (1.68–1.85)	<0.001
Medicare/Medicaid plus private insurance	1.39 (1.31–1.48)	<0.001	1.44 (1.37–1.52)	<0.001	1.47 (1.40–1.55)	<0.001
Self-Pay/Safety net	1.01 (0.86–1.18)	0.903	0.96 (0.84–1.11)	0.6	1.01 (0.88–1.14)	0.934
Military or government	1.12 (0.89–1.41)	0.35	1.23 (1.01–1.49)	0.036	1.23 (1.02–1.47)	0.029
COVID pay	0.69 (0.56–0.85)	0.001	0.52 (0.42–0.63)	<0.001	0.46 (0.38–0.56)	<0.001
Other	0.99 (0.70–1.39)	0.943	1.04 (0.78–1.39)	0.772	1.00 (0.76–1.32)	0.985
Unknown	1.37 (1.29–1.45)	<0.001	1.40 (1.33–1.47)	<0.001	1.41 (1.34–1.47)	<0.001

Children under 18, pregnant patients, and patients who expired at their index hospitalization were excluded from readmission analyses.NH, Non-Hispanic; OR, odds ratio; CI, confidence interval.

**Table 3:** Multivariable logistic regression: readmission among patients with a COVID-19-associated inpatient hospitalization.

Multivariable logistic regression, *N* = 91,007	30 day readmission		60 day readmission		90 day readmission	
Events	*n* = 11,912		*N* = 16,865		*N* = 19,679	
	*aOR (95% CI)*	*p value*	*aOR (95% CI)*	*p value*	*aOR (95% CI)*	*p value*
Age (years)						
18–29	REF		REF		REF	
30–49	1.04 (0.95–1.13)	0.439	1.01 (0.93–1.09)	0.873	1.00 (0.93–1.08)	0.971
50–69	1.24 (1.14–1.35)	<0.001	1.20 (1.11–1.29)	<0.001	1.16 (1.08–1.25)	<0.001
70+	1.44 (1.32–1.57)	<0.001	1.42 (1.32–1.54)	<0.001	1.40 (1.30–1.50)	<0.001
Sex						
Male	REF		REF		REF	
Female, non-pregnant	0.99 (0.95–1.03)	0.609	1.03 (1.00–1.07)	0.071	1.06 (1.02–1.09)	0.001
Race/Ethnicity						
Non-Hispanic White	REF		REF		REF	
Non-Hispanic Black	1.06 (1.00–1.11)	0.047	1.08 (1.04–1.14)	<0.001	1.07 (1.02–1.12)	0.002
Non-Hispanic Asian	0.89 (0.79–1.01)	0.075	0.82 (0.73–0.92)	<0.001	0.77 (0.69–0.86)	<0.001
Non-Hispanic American Indian/Alaska Native	0.89 (0.65–1.24)	0.497	0.80 (0.60–1.07)	0.133	0.83 (0.63–1.10)	0.194
Non-Hispanic Native Hawaiian/ Pacific Islander	1.22 (0.79–1.90)	0.369	1.22 (0.83–1.80)	0.313	1.11 (0.76–1.62)	0.589
Non-Hispanic Other	0.85 (0.77–0.93)	<0.001	0.80 (0.74–0.86)	<0.001	0.79 (0.73–0.85)	<0.001
Hispanic	0.87 (0.83–0.92)	<0.001	0.82 (0.79–0.86)	<0.001	0.80 (0.77–0.84)	<0.001
Missing	0.15 (0.07–0.30)	<0.001	0.11 (0.06–0.21)	<0.001	0.13 (0.07–0.23)	<0.001
Area deprivation index						
Quintile 1 (Least Disadvantaged)	REF		REF		REF	
Quintile 2	1.02 (0.95–1.09)	0.612	1.06 (1.00–1.13)	0.035	1.08 (1.02–1.14)	0.005
Quintile 3	1.08 (1.01–1.15)	0.023	1.12 (1.05–1.18)	<0.001	1.12 (1.06–1.18)	<0.001
Quintile 4	1.12 (1.05–1.20)	0.001	1.15 (1.08–1.22)	<0.001	1.17 (1.11–1.24)	<0.001
Quintile 5 (Most Disadvantaged)	1.11 (1.04–1.20)	0.003	1.14 (1.07–1.21)	<0.001	1.16 (1.09–1.24)	<0.001
Charlson Comorbidity Index	1.19 (1.17–1.20)	<0.001	1.21 (1.20–1.22)	<0.001	1.23 (1.22–1.24)	<0.001
Date of index hospitalization						
February 1, 2020- September 15, 2020	REF		REF		REF	
September 16, 2020- June 20, 2021	0.88 (0.83–0.93)	<0.001	0.91 (0.86–0.96)	<0.001	0.91 (0.86–0.95)	<0.001
June 21, 2021- November 20, 2021	0.77 (0.72–0.83)	<0.001	0.79 (0.75–0.84)	<0.001	0.78 (0.74–0.82)	<0.001
November 21, 2021- April 15, 2022	1.09 (1.01–1.16)	0.018	1.19 (1.12–1.27)	<0.001	1.23 (1.16–1.30)	<0.001
April 16, 2022- November 30, 2022	1.03 (0.96–1.11)	0.391	1.17 (1.10–1.25)	<0.001	1.12 (1.05–1.19)	<0.001
Length of stay (index hospitalization)						
<2 days	2.31 (2.17–2.46)	<0.001	2.12 (2.00–2.24)	<0.001	1.98 (1.88–2.09)	<0.001
2–3 days	1.22 (1.14–1.30)	<0.001	1.14 (1.07–1.20)	<0.001	1.11 (1.05–1.17)	<0.001
4–5 days	REF		REF		REF	
6–7 days	0.98 (0.91–1.06)	0.616	1.03 (0.96–1.10)	0.439	1.06 (0.98–1.14)	0.346
8–9 days	1.08 (0.99–1.18)	0.101	1.09 (1.01–1.18)	0.031	1.06 (0.98–1.14)	0.152
10+ days	1.02 (0.96–1.09)	0.542	1.10 (1.04–1.16)	0.001	1.06 (1.00–1.11)	0.040
Financial class (index hospitalization)						
Private insurance	REF		REF		REF	
Medicare/Medicaid alone	1.31 (1.23–1.39)	<0.001	1.34 (1.27–1.42)	<0.001	1.38 (1.31–1.46)	<0.001
Medicare/Medicaid plus private insurance	1.19 (1.11–1.26)	<0.001	1.20 (1.14–1.27)	<0.001	1.22 (1.16–1.28)	<0.001
Self-Pay/Safety net	1.05 (0.90–1.24)	0.521	1.02 (0.89–1.18)	0.751	1.07 (0.93–1.22)	0.33
Military or government	0.99 (0.78–1.26)	0.951	1.05 (0.86–1.28)	0.637	1.05 (0.87–1.27)	0.621
COVID pay	0.94 (0.76–1.17)	0.578	0.73 (0.60–0.90)	0.003	0.65 (0.54–0.80)	<0.001
Other	0.98 (0.66–1.47)	0.942	1.08 (0.77–1.52)	0.639	1.07 (0.78–1.47)	0.691
Unknown	1.27 (1.19–1.34)	<0.001	1.28 (1.21–1.35)	<0.001	1.28 (1.22–1.35)	<0.001

Children under 18, pregnant patients, and patients who expired at their index hospitalization were excluded from readmission analyses. 30 day readmission model area under the receiver operating characteristic curve: 0.6539; 60 day readmission model area under the curve: 0.6648; 90 day readmission model area under the curve: 0.6692.NH, Non-Hispanic; aOR, adjusted odds ratio; CI, confidence interval.

## 4 Discussion

In this cohort of 91,007 adult patients with a COVID-19-associated hospitalization, over 21% were readmitted to the hospital within 90 days of their initial visit (*n* = 19,679), and 13% were readmitted within the first 30 days (*n* = 11,912). While this study did not seek to determine the cause of admission or readmission, the relative frequencies of diagnoses such as pneumonia, acute respiratory failure, and hypoxia are characteristics of a population of patients with severe COVID-19. Total 30-day readmission risk varied significantly across time points, falling during the period of July 2021 through November 2021, when Delta was the dominant circulating variant and then peaking during the period of December 2021 to December 2022, as Omicron group variants became dominant.

The measured social determinants of health, including race/ethnicity, relative neighborhood disadvantage (ADI), and insurance status, were all associated with readmission risk. Non-Hispanic Black patients were more likely to be readmitted at 30, 60, and 90 days, while Hispanic patients were less likely to be readmitted at all time points, compared to non-Hispanic White patients. However, when mortality and readmission were considered as a composite outcome, Hispanic patients were not at greater risk, which may indicate a survivorship bias among certain subgroups. Likewise, increasing neighborhood disadvantage displayed a clear dose–response relationship to readmission in age-adjusted time-to-event analysis and logistic regression models. While communities of color bore disproportionate COVID-19-related mortality early in the pandemic (32, 33), the demographic proportions of COVID-19 cases, hospitalizations, and deaths have varied widely across each wave of the pandemic (1). The observed associations between race, ethnicity, socioeconomic status, and poor health outcomes are unlikely biological in origin. As COVID-19 transitions into an endemic condition, further research is needed to elucidate the specific barriers to accessing quality, timely care for COVID-19 and to develop interventions to curb preventable readmissions within vulnerable communities.

The readmission rate demonstrated in this study is high relative to the extant literature, especially given the proportion of patients under 50 years of age (34%; 31,267/91,007) and patients with a zero score on the Charlson Comorbidity Index (47%; 42,413/91,007) (34, 35). This gap could be explained by the capacity of the health information exchange to identify encounters across institutions and hospital systems: 26% of readmission encounters were to a different hospital or hospital system from the index hospitalization encounter. Increasing length of stay is often used as a proxy of disease severity at the index encounter (36, 37), but in this study, the length of stay of the index encounter displayed a parabolic effect: readmission risk was highest in patients whose index encounter was either less than 2 days or 10 or more days. These results suggest some patients may have either been discharged prematurely or decompensated quickly after transitioning to outpatient care, possibly due to overburdened hospital and primary care facilities during epidemic peaks.

The breadth and depth of the HIE data facilitated accurate patient tracking across time and between facilities and enabled investigators to correctly determine readmission status, regardless of whether patients returned to the same hospital. Our analyses are further strengthened by the addition of neighborhood-level measures of disadvantage and encounter-specific insurance information. As we utilized neighborhood-level socioeconomic measures that have been normalized across United States national and state populations, our findings will be valuable in comparative analyses across regions. We chose to exclude pregnant patients from readmission analyses, as they likely represent a population of incidentally captured subclinical COVID-19 cases who are inherently at high risk for readmission. However, future studies are needed to investigate COVID-19-related maternal and fetal outcomes, as well as healthcare utilization among pregnant COVID-19 patients. The primary outcome was all-cause readmission; patients with readmissions due to causes unrelated to COVID-19 were likely included in this analysis. Additionally, due to a high number of index encounters with missing discharge disposition data, we analyzed readmission risk for living patients irrespective of discharge status, which may have resulted in the misclassification of some transfer encounters as readmissions. However, the proportion of transferred patients was relatively low (<7%) and consistent with other studies in the region (38, 39). As with all EHR-based research, events occurring outside of the healthcare system, including death outside of a hospital facility, are challenging to collect. While we were able to collect date of death from some patients who expired in the community, some patients who died after leaving their index encounter may have been classified as non-readmissions. Despite these limitations, our study adds to the growing body of evidence characterizing social determinants of COVID-19 healthcare utilization and disease outcomes throughout 3 years of the pandemic.

More than 20% of patients in this large, HIE-based cohort with a COVID-19-associated hospitalization were readmitted within 90 days of their index encounter, demonstrating the lasting impact of COVID-19 infection, especially among members of marginalized communities, and the increasing burden of COVID-19 morbidity on the healthcare system. Multiple investigations throughout the pandemic reported COVID-19 patients suffering substantial and long-lasting health changes, including decreased respiratory and cardiovascular function, ongoing symptoms requiring clinical intervention, and decreased quality of life in the months or even years following even apparently mild COVID-19 episodes (40–42). Our findings further illustrate the ongoing changes in patients’ experiences of COVID-19 over 3 years of the pandemic and emphasize the need for transitional care for COVID-19 patients leaving the hospital. As growing numbers face the specter of long COVID, health authorities must ensure all patients have access to quality care, build trust in the health system among vulnerable populations, and ensure institutions have the capacity to provide care in the post-acute period.

## Data availability statement

The datasets presented in this article are not readily available because clinical data cannot be shared publicly because of patient confidentiality concerns as imposed by the University of Texas Health Science Center at Houston Committee for the Protection of Human Subjects. Requests to access de-identified data can be made to cphs@uth.tmc.edu which will be evaluated on a case by case basis in line with institutional policies. Requests to access the datasets should be directed to Committee for the Protection of Human Subjects: cphs@uth.tmc.edu.

## Ethics statement

The studies involving humans were approved by the Western Institutional Review Board as a quality improvement study and granted a waiver of informed consent. The studies were conducted in accordance with the local legislation and institutional requirements. Written informed consent for participation was not required from the participants or the participants’ legal guardians/next of kin in accordance with the national legislation and institutional requirements.

## Author contributions

MS: Writing – review & editing, Writing – original draft, Visualization, Formal analysis, Conceptualization. JM: Writing – review & editing, Validation, Data curation. MF: Writing – review & editing, Validation, Data curation. GT: Writing – review & editing, Validation, Data curation, Conceptualization. TC: Writing – review & editing, Supervision, Methodology, Formal analysis. RR: Writing – review & editing, Formal analysis. JH: Writing – review & editing, Supervision, Resources. EB: Writing – review & editing, Writing – original draft, Supervision, Resources, Conceptualization.

## Footnotes

^1^ https://dshs.texas.gov/coronavirus
