# Supplementary material for: Social determinants of health predict readmission following COVID-19 hospitalization: a health information exchange-based retrospective cohort study
Source: Front Public Health. 2024 Mar 27;12:1352240. doi: 10.3389/fpubh.2024.1352240 (PMC11004289; doi:10.3389/fpubh.2024.1352240)
Supplement: Supplementary file 2 [file Data_Sheet_2.docx]

Supplement 2

| Supplemental Table 1: Diagnosis frequencies within index hospitalizations | |
| --- | --- |
|  | Total |
|  | N=88,041 |
| **Patients with diagnosis** | *n (%)* |
| COVID-19 | 77,462 (88.0%) |
| Pneumonia | 46,035 (52.3%) |
| Acute respiratory failure | 35,758 (40.6%) |
| Hypoxemia or hypoxia | 34,021 (38.6%) |
| Hypertension | 24,199 (27.5%) |
| Diabetes | 14,941 (17.0%) |
| Thrombosis | 14,847 (16.9%) |
| Embolism | 14,467 (16.4%) |
| Shortness of breath | 13,986 (15.9%) |
| Renal failure | 13,237 (15.0%) |
| Sepsis | 12,128 (13.8%) |
| Acute kidney injury | 10,965 (12.5%) |
| Chronic kidney disease | 9,303 (10.6%) |
| Note: Diagnosis data were available for 88,041 out of 104,196 index encounters (84%) | |
|  |  |

| Supplemental Table 2: Diagnosis frequencies within 90-day readmission hospitalizations | |
| --- | --- |
|  | Total |
|  | N=18,416 |
| **Patients with diagnosis** | *n (%)* |
| Hypertension | 6,322 (34.3%) |
| COVID-19 | 5,982 (32.5%) |
| Acute respiratory failure | 5,054 (27.4%) |
| Pneumonia | 4,960 (26.9%) |
| Hypoxemia or hypoxia | 4,294 (23.3%) |
| Diabetes | 3,817 (20.7%) |
| Renal failure | 3,404 (18.5%) |
| Heart failure | 3,390 (18.4%) |
| Chronic kidney disease | 3,322 (18.0%) |
| Shortness of breath | 2,883 (15.7%) |
| End stage renal disease | 2,573 (14.0%) |
| Acute kidney injury | 2,557 (13.9%) |
| Sepsis | 2,538 (13.8%) |
| Trauma | 2,522 (13.7%) |
| Urinary tract infection | 2,218 (12.0%) |
| Thrombosis | 1,864 (10.1%) |
| Note: Diagnosis data were available for 18,416 out of 20,606 index encounters (89%) | |
|  |  |
