# Supplementary material for: Social determinants of health predict readmission following COVID-19 hospitalization: a health information exchange-based retrospective cohort study
Source: Front Public Health. 2024 Mar 27;12:1352240. doi: 10.3389/fpubh.2024.1352240 (PMC11004289; doi:10.3389/fpubh.2024.1352240)

Supplement 3- Race/ethnicity and readmission risk

| **Supplemental Table 3.1: Cohort characteristics, stratified by epidemic peak** | | | | | | |
| --- | --- | --- | --- | --- | --- | --- |
| Cohort characteristics | February 1, 2020- September 15, 2020, N=18,343 | September 16, 2020- June 20, 2021, N=34,919 | June 21, 2021- November 20, 2021, N=23,319 | November 21, 2021- April 15, 2022, N=15,267 | April 16, 2022- November 30, 2022, N=12,344 |  |
|  | *n (%)* | *n (%)* | *n (%)* | *n (%)* | *n (%)* |  |
| Age (years) |  |  |  |  |  |  |
| under 18 | 482 ( 2.6%) | 1,263 ( 3.6%) | 642 ( 2.8%) | 303 ( 2.0%) | 345 ( 2.8%) |  |
| 18-29 | 2,069 (11.3%) | 3,341 ( 9.6%) | 2,117 ( 9.1%) | 1,471 ( 9.6%) | 947 ( 7.7%) |  |
| 30-49 | 4,928 (26.9%) | 9,374 (26.8%) | 7,329 (31.4%) | 3,143 (20.6%) | 1,928 (15.6%) |  |
| 50-69 | 6,488 (35.4%) | 12,699 (36.4%) | 8,409 (36.1%) | 5,155 (33.8%) | 3,767 (30.5%) |  |
| 70+ | 4,376 (23.9%) | 8,242 (23.6%) | 4,822 (20.7%) | 5,195 (34.0%) | 5,354 (43.4%) |  |
| Sex |  |  |  |  |  |  |
| Male | 8,451 (46.1%) | 16,627 (47.7%) | 11,867 (50.9%) | 6,983 (45.8%) | 5,561 (45.2%) |  |
| Female, pregnant | 737 ( 4.0%) | 1,049 ( 3.0%) | 845 ( 3.6%) | 1,125 ( 7.4%) | 778 ( 6.3%) |  |
| Female, non-pregnant | 9,139 (49.9%) | 17,204 (49.3%) | 10,592 (45.5%) | 7,123 (46.8%) | 5,965 (48.5%) |  |
| Race/Ethnicity |  |  |  |  |  |  |
| Non-Hispanic White | 6,141 (33.8%) | 14,905 (43.1%) | 11,004 (47.6%) | 7,469 (49.3%) | 6,547 (53.4%) |  |
| Non-Hispanic Black | 4,027 (22.1%) | 6,256 (18.1%) | 4,025 (17.4%) | 3,065 (20.2%) | 2,291 (18.7%) |  |
| Non-Hispanic Asian | 517 ( 2.8%) | 1,115 ( 3.2%) | 420 ( 1.8%) | 432 ( 2.8%) | 443 ( 3.6%) |  |
| Non-Hispanic American Indian/Alaska Native | 50 ( 0.3%) | 144 ( 0.4%) | 71 ( 0.3%) | 53 ( 0.3%) | 53 ( 0.4%) |  |
| Non-Hispanic Native Hawaiian/ Pacific Islander | 25 ( 0.1%) | 58 ( 0.2%) | 47 ( 0.2%) | 35 ( 0.2%) | 25 ( 0.2%) |  |
| Non-Hispanic Other | 1,421 ( 7.8%) | 2,520 ( 7.3%) | 1,503 ( 6.5%) | 592 ( 3.9%) | 584 ( 4.8%) |  |
| Hispanic | 5,968 (32.8%) | 9,494 (27.4%) | 5,952 (25.7%) | 3,392 (22.4%) | 2,233 (18.2%) |  |
| Missing | 42 ( 0.2%) | 130 ( 0.4%) | 99 ( 0.4%) | 124 ( 0.8%) | 87 ( 0.7%) |  |
| Area deprivation index |  |  |  |  |  |  |
| Quintile 1 (Least Disadvantaged) | 2,497 (13.8%) | 5,715 (16.5%) | 3,662 (15.8%) | 2,735 (18.1%) | 2,552 (20.8%) |  |
| Quintile 2 | 3,452 (19.0%) | 7,629 (22.0%) | 5,381 (23.2%) | 3,283 (21.7%) | 2,668 (21.7%) |  |
| Quintile 3 | 4,489 (24.8%) | 8,638 (24.9%) | 5,612 (24.2%) | 3,612 (23.8%) | 2,774 (22.6%) |  |
| Quintile 4 | 4,627 (25.5%) | 7,833 (22.6%) | 5,008 (21.6%) | 3,306 (21.8%) | 2,599 (21.2%) |  |
| Quintile 5 (Most Disadvantaged) | 3,064 (16.9%) | 4,920 (14.2%) | 3,526 (15.2%) | 2,216 (14.6%) | 1,675 (13.7%) |  |
| Charlson Comorbidity Index | 0 (0-1) | 0 (0-1) | 0 (0-1) | 1 (0-2) | 1 (0-2) |  |
| Length of stay (index hospitalization) |  |  |  |  |  |  |
| <2 days | 1,233 ( 6.7%) | 8,708 (24.9%) | 4,517 (19.4%) | 2,577 (16.9%) | 1,801 (14.6%) |  |
| 2-3 days | 4,038 (22.0%) | 6,951 (19.9%) | 4,153 (17.8%) | 3,842 (25.2%) | 3,628 (29.4%) |  |
| 4-5 days | 3,660 (20.0%) | 6,474 (18.5%) | 4,047 (17.4%) | 3,016 (19.8%) | 2,885 (23.4%) |  |
| 6-7 days | 2,373 (12.9%) | 3,555 (10.2%) | 2,893 (12.4%) | 1,892 (12.4%) | 1,469 (11.9%) |  |
| 8-9 days | 1,436 ( 7.8%) | 1,982 ( 5.7%) | 1,752 ( 7.5%) | 1,063 ( 7.0%) | 796 ( 6.4%) |  |
| 10+ days | 5,603 (30.5%) | 7,249 (20.8%) | 5,957 (25.5%) | 2,877 (18.8%) | 1,765 (14.3%) |  |
| Financial class (index hospitalization) |  |  |  |  |  |  |
| Private insurance | 3,287 (17.9%) | 8,007 (22.9%) | 5,681 (24.4%) | 2,890 (18.9%) | 1,863 (15.1%) |  |
| Medicare/Medicaid alone | 4,896 (26.7%) | 8,185 (23.4%) | 5,075 (21.8%) | 4,276 (28.0%) | 3,109 (25.2%) |  |
| Medicare/Medicaid plus private insurance | 3,692 (20.1%) | 8,373 (24.0%) | 5,111 (21.9%) | 3,581 (23.5%) | 2,775 (22.5%) |  |
| Self-Pay/Safety net | 388 ( 2.1%) | 677 ( 1.9%) | 452 ( 1.9%) | 301 ( 2.0%) | 223 ( 1.8%) |  |
| Military or government | 109 ( 0.6%) | 294 ( 0.8%) | 210 ( 0.9%) | 125 ( 0.8%) | 105 ( 0.9%) |  |
| COVID pay | 38 ( 0.2%) | 439 ( 1.3%) | 888 ( 3.8%) | 106 ( 0.7%) | 8 ( 0.1%) |  |
| Other | 111 ( 0.6%) | 153 ( 0.4%) | 89 ( 0.4%) | 56 ( 0.4%) | 27 ( 0.2%) |  |
| Unknown | 5,822 (31.7%) | 8,791 (25.2%) | 5,813 (24.9%) | 3,932 (25.8%) | 4,231 (34.3%) |  |
| Stratification by date of index COVID-associated hospitalization | | | | | | |
|  |  |  |  |  |  |  |

| **Supplemental Table 3.2: Multivariate Cox regression: 90-day readmission among patients with a COVID-associated inpatient hospitalization, stratified by epidemic peak** | | | | | | | | | | |
| --- | --- | --- | --- | --- | --- | --- | --- | --- | --- | --- |
| Multivariate time-to-event regression | February 1, 2020- September 15, 2020, N=15,858 | | September 16, 2020- June 20, 2021, N=30,630 | | June 21, 2021- November 20, 2021, N=19,180 | | November 21, 2021- April 15, 2022, N=12,654 | | April 16, 2022- November 30, 2022, N=10,584 | |
| **Events** | n=3,347 | | n=6,318 | | n=3,388 | | n=3,703 | | n=2,922 | |
|  | *HR (95% CI)* | *p-value* | *HR (95% CI)* | *p-value* | *HR (95% CI)* | *p-value* | *HR (95% CI)* | *p-value* | *HR (95% CI)* | *p-value* |
| Age (years) |  |  |  |  |  |  |  |  |  |  |
| 18-29 | REF |  | REF |  | REF |  | REF |  | REF |  |
| 30-49 | 0.9 (0.8-1.1) | 0.313 | 1.0 (0.9-1.1) | 0.664 | 1.1 (0.9-1.3) | 0.206 | 1.3 (1.1-1.5) | 0.006 | 1.1 (0.9-1.4) | 0.362 |
| 50-69 | 1.0 (0.9-1.2) | 0.713 | 1.0 (0.9-1.2) | 0.381 | 1.6 (1.3-1.8) | <0.001 | 1.4 (1.2-1.7) | <0.001 | 1.2 (1.0-1.5) | 0.056 |
| 70+ | 1.2 (1.0-1.3) | 0.027 | 1.3 (1.2-1.5) | <0.001 | 2.1 (1.8-2.5) | <0.001 | 1.5 (1.2-1.7) | <0.001 | 1.1 (0.9-1.4) | 0.208 |
| Sex |  |  |  |  |  |  |  |  |  |  |
| Male | REF |  | REF |  | REF |  | REF |  | REF |  |
| Female, non-pregnant | 1.1 (1.0-1.1) | 0.068 | 1.1 (1.0-1.1) | 0.019 | 1.1 (1.0-1.2) | 0.007 | 1.0 (0.9-1.0) | 0.508 | 0.9 (0.8-1.0) | 0.01 |
| Race/Ethnicity |  |  |  |  |  |  |  |  |  |  |
| Non-Hispanic White | REF |  | REF |  | REF |  | REF |  | REF |  |
| Non-Hispanic Black | 1.0 (0.9-1.1) | 0.377 | 1.0 (1.0-1.1) | 0.228 | 1.1 (1.0-1.2) | 0.134 | 1.1 (1.0-1.2) | 0.134 | 1.2 (1.1-1.3) | <0.001 |
| Non-Hispanic Asian | 0.7 (0.6-0.9) | 0.012 | 0.7 (0.6-0.9) | 0.001 | 0.8 (0.6-1.1) | 0.234 | 1.0 (0.9-1.3) | 0.64 | 0.8 (0.7-1.1) | 0.138 |
| Non-Hispanic American Indian/Alaska Native | 0.7 (0.4-1.4) | 0.342 | 1.2 (0.6-0.9) | 0.416 | 1.1 (0.6-1.9) | 0.716 | 0.5 (0.3-1.1) | 0.096 | 0.6 (0.3-1.2) | 0.177 |
| Non-Hispanic Native Hawaiian/ Pacific Islander | 0.6 (0.2-1.9) | 0.397 | 1.3 (0.8-2.3) | 0.303 | 1.9 (1.0-3.5) | 0.044 | 0.8 (0.3-2.0) | 0.667 | 0.5 (0.2-1.7) | 0.287 |
| Non-Hispanic Other | 0.6 (0.6-0.8) | <0.001 | 0.8 (0.7-0.9) | <0.001 | 0.8 (0.6-0.9) | 0.001 | 1.0 (0.8-1.2) | 0.827 | 1.1 (0.9-1.3) | 0.302 |
| Hispanic | 0.7 (0.6-0.7) | <0.001 | 0.8 (0.8-0.9) | <0.001 | 0.9 (0.8-1.0) | 0.003 | 1.0 (0.9-1.0) | 0.347 | 1.0 (0.9-1.2) | 0.477 |
| Missing | 0.1 (0.1-0.2) | 0.029 | 0.1 (0.0-0.4) | <0.001 | 0.1 (0.0-0.8) | 0.025 | 0.1 (0.0-0.4) | <0.001 | 0.3 (0.1-0.7) | 0.008 |
| Area deprivation index |  |  |  |  |  |  |  |  |  |  |
| Quintile 1 (Least Disadvantaged) | REF |  | REF |  | REF |  | REF |  | REF |  |
| Quintile 2 | 1.1 (0.9-1.2) | 0.263 | 1.1 (1.0-1.2) | 0.072 | 1.1 (0.9-1.2) | 0.345 | 1.0 (0.9-1.2) | 0.401 | 1.0 (0.9-1.2) | 0.492 |
| Quintile 3 | 1.1 (1.0-1.2) | 0.168 | 1.2 (1.1-1.3) | 0.001 | 1.1 (1.0-1.3) | 0.043 | 1.1 (1.0-1.2) | 0.091 | 1.0 (0.9-1.1) | 0.622 |
| Quintile 4 | 1.1 (1.0-1.3) | 0.09 | 1.2 (1.1-1.3) | <0.001 | 1.1 (1.0-1.2) | 0.109 | 1.2 (1.0-1.3) | 0.01 | 1.1 (0.9-1.2) | 0.381 |
| Quintile 5 (Most Disadvantaged) | 1.1 (1.0-1.3) | 0.081 | 1.2 (1.1-1.3) | 0.002 | 1.2 (1.1-1.4) | 0.001 | 1.1 (1.0-1.3) | 0.026 | 1.0 (0.9-1.2) | 0.577 |
| Charlson Comorbidity Index | 1.1 (1.1-1.2) | <0.001 | 1.2 (1.2-1.2) | <0.001 | 1.2 (1.2-1.2) | <0.001 | 1.2 (1.1-1.2) | <0.001 | 1.1 (1.1-1.1) | <0.001 |
| Length of stay (index hospitalization) |  |  |  |  |  |  |  |  |  |  |
| <2 days | 2.6 (2.2-2.9) | <0.001 | 1.8 (1.7-1.9) | <0.001 | 2.4 (2.2-2.7) | <0.001 | 1.6 (1.4-1.7) | <0.001 | 1.4 (1.3-1.6) | <0.001 |
| 2-3 days | 1.4 (1.2-1.5) | <0.001 | 1.3 (1.2-1.4) | <0.001 | 1.3 (1.1-1.4) | <0.001 | 0.8 (0.7-0.9) | 0.001 | 0.8 (0.7-0.9) | 0.001 |
| 4-5 days | REF |  | REF |  | REF |  | REF |  | REF |  |
| 6-7 days | 1.2 (1.0-1.3) | 0.023 | 0.9 (0.8-1.0) | 0.12 | 0.9 (0.8-1.1) | 0.284 | 1.1 (0.9-1.2) | 0.369 | 1.1 (1.0-1.2) | 0.167 |
| 8-9 days | 1.2 (1.0-1.4) | 0.043 | 1.0 (0.9-1.1) | 0.912 | 0.9 (0.8-1.1) | 0.323 | 1.1 (1.0-1.3) | 0.158 | 1.2 (1.0-1.4) | 0.064 |
| 10+ days | 1.0 (0.9-1.1) | 0.968 | 1.0 (0.9-1.1) | 0.835 | 1.0 (0.9-1.1) | 0.962 | 1.1 (1.0-1.2) | 0.227 | 1.3 (1.1-1.4) | <0.001 |
| Financial class (index hospitalization) |  |  |  |  |  |  |  |  |  |  |
| Private insurance | REF |  | REF |  | REF |  | REF |  | REF |  |
| Medicare/Medicaid alone | 1.3 (1.1-1.4) | <0.001 | 1.3 (1.2-1.4) | <0.001 | 1.6 (1.4-1.7) | <0.001 | 1.3 (1.2-1.5) | <0.001 | 1.0 (0.9-1.1) | 0.877 |
| Medicare/Medicaid plus private insurance | 1.2 (1.1-1.4) | 0.001 | 1.2 (1.1-1.3) | <0.001 | 1.3 (1.2-1.5) | <0.001 | 1.3 (1.2-1.4) | <0.001 | 0.9 (0.8-1.1) | 0.398 |
| Self-Pay/Safety net | 1.0 (0.8-1.3) | 0.907 | 1.2 (1.0-1.4) | 0.109 | 1.0 (0.7-1.4) | 0.826 | 1.2 (0.9-1.6) | 0.222 | 0.6 (0.4-0.9) | 0.005 |
| Military or government | 0.6 (0.3-1.1) | 0.099 | 1.1 (0.8-1.5) | 0.479 | 1.1 (0.7-1.6) | 0.782 | 1.5 (1.0-2.1) | 0.038 | 0.9 (0.5-1.3) | 0.51 |
| COVID pay | 0.4 (0.1-1.3) | 0.126 | 0.5 (0.3-0.7) | <0.001 | 1.2 (0.9-1.5) | 0.202 | 0.5 (0.2-0.9) | 0.02 | 0.6 (0.1-4.1) | 0.582 |
| Other | 0.7 (0.3-1.6) | 0.419 | 1.2 (0.8-1.8) | 0.351 | 0.8 (0.9-1.5) | 0.686 | 1.1 (0.5-2.2) | 0.818 | 0.3 (0.0-1.9) | 0.194 |
| Unknown | 1.1 (0.9-1.2) | 0.316 | 1.2 (1.1-1.3) | <0.001 | 1.5 (1.4-1.7) | <0.001 | 1.2 (1.1-1.4) | <0.001 | 1.1 (1.0-1.2) | 0.264 |
|  | AUC: 0.6363 | | AUC: 0.6456 | | AUC: 0.6947 | | AUC: 0.6286 | | AUC: 0.6094 | |
| Note: Children under 18, pregnant patients, and patients who expired at their index hospitalization were excluded from readmission analyses.  Stratification by date of index COVID-associated hospitalization | | | | | | | | | | |
|  |  |  |  |  |  |  |  |  |  |  |

| **Supplemental Table 3.3: Multivariable logistic regression: composite 30-day readmission or death among patients with a COVID-associated inpatient hospitalization** | | |
| --- | --- | --- |
| Multivariable logistic regression | 30 day readmission or death, N=95,275 | |
| **Events** | n=18,652 | |
|  | *OR (95% CI)* | *p-value* |
| Age (years) |  |  |
| 18-29 | REF |  |
| 30-49 | 1.1 (1.0-1.2) | 0.005 |
| 50-69 | 1.5 (1.4-1.7) | <0.001 |
| 70+ | 2.2 (2.0-2.4) | <0.001 |
| Sex |  |  |
| Male | REF |  |
| Female, non-pregnant | 0.9 (0.9-0.9) | <0.001 |
| Race/Ethnicity |  |  |
| Non-Hispanic White | REF |  |
| Non-Hispanic Black | 1.1 (1.0-1.1) | 0.002 |
| Non-Hispanic Asian | 1.0 (0.9-1.1) | 0.435 |
| Non-Hispanic American Indian/Alaska Native | 0.9 (0.7-1.2) | 0.712 |
| Non-Hispanic Native Hawaiian/ Pacific Islander | 1.2 (0.8-1.7) | 0.366 |
| Non-Hispanic Other | 0.9 (0.9-1.0) | 0.026 |
| Hispanic | 1.0 (0.9-1.0) | 0.078 |
| Missing | 0.7 (0.6-1.0) | 0.039 |
| Area deprivation index |  |  |
| Quintile 1 (Least Disadvantaged) | REF |  |
| Quintile 2 | 1.0 (1.0-1.1) | 0.183 |
| Quintile 3 | 1.1 (1.0-1.1) | 0.008 |
| Quintile 4 | 1.1 (1.1-1.2) | <0.001 |
| Quintile 5 (Most Disadvantaged) | 1.1 (1.1-1.2) | <0.001 |
| Charlson Comorbidity Index | 1.1 (1.1-1.2) | <0.001 |
| Date of index hospitalization |  |  |
| February 1, 2020- September 15, 2020 | REF |  |
| September 16, 2020- June 20, 2021 | 0.9 (0.9-1.0) | 0.001 |
| June 21, 2021- November 20, 2021 | 1.2 (1.1-1.3) | <0.001 |
| November 21, 2021- April 15, 2022 | 1.3 (1.2-1.3) | <0.001 |
| April 16, 2022- November 30, 2022 | 1.1 (1.0-1.2) | 0.007 |
| Length of stay (index hospitalization) |  |  |
| <2 days | 2.4 (2.3-2.6) | <0.001 |
| 2-3 days | 1.2 (1.2-1.3) | <0.001 |
| 4-5 days | REF |  |
| 6-7 days | 1.1 (1.0-1.2) | 0.009 |
| 8-9 days | 1.4 (1.3-1.5) | <0.001 |
| 10+ days | 1.8 (1.7-1.9) | <0.001 |
| Financial class (index hospitalization) |  |  |
| Private insurance | REF |  |
| Medicare/Medicaid alone | 1.3 (1.2-1.4) | <0.001 |
| Medicare/Medicaid plus private insurance | 1.2 (1.1-1.3) | <0.001 |
| Self-Pay/Safety net | 1.3 (1.1-1.4) | <0.001 |
| Military or government | 1.1 (0.9-1.3) | 0.344 |
| COVID pay | 1.3 (1.3-1.6) | <0.001 |
| Other | 1.8 (1.4-2.4) | <0.001 |
| Unknown | 1.4 (1.3-1.4) | <0.001 |
| Note: Children under 18 and pregnant patients were excluded from readmission/mortality analyses.  30 day composite outcome model area under the curve: 0.6613 | | |
|  |  |  |

**Supplemental Figure 3.1**: 30-day Readmission Risk Over Time by Race and Ethnicity


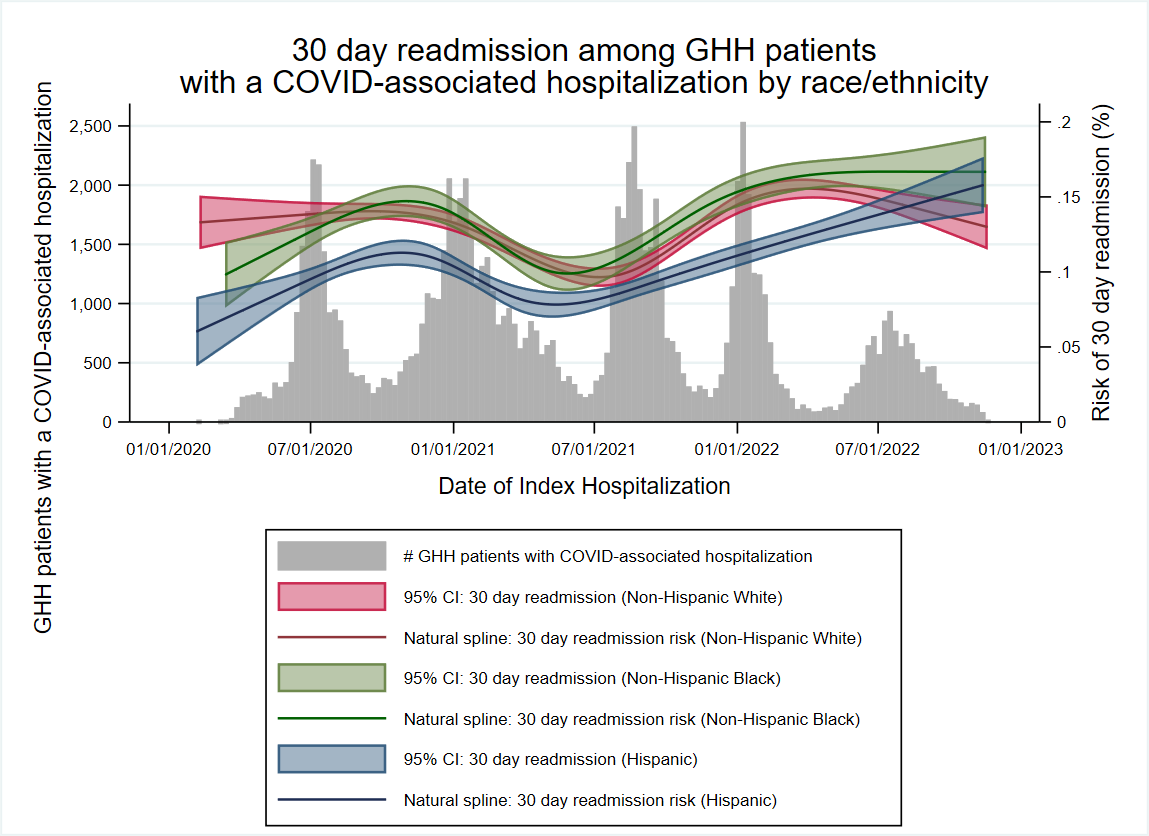

Supplement: Supplementary file 3 [file Data_Sheet_3.docx]
