# Supplementary figures and images for: Social determinants of health predict readmission following COVID-19 hospitalization: a health information exchange-based retrospective cohort study
Source: Front Public Health. 2024 Mar 27;12:1352240. doi: 10.3389/fpubh.2024.1352240 (PMC11004289; doi:10.3389/fpubh.2024.1352240)

Supplemental Figure 4.1


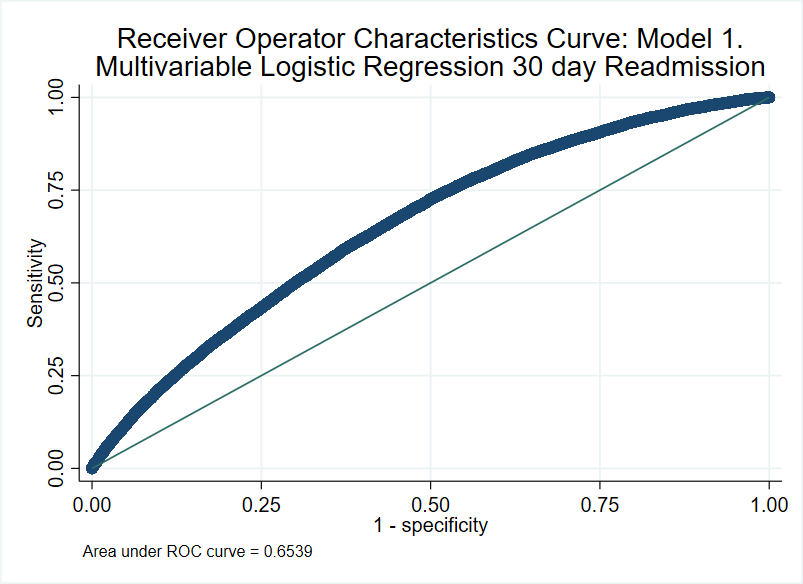


Supplemental Figure 4.2
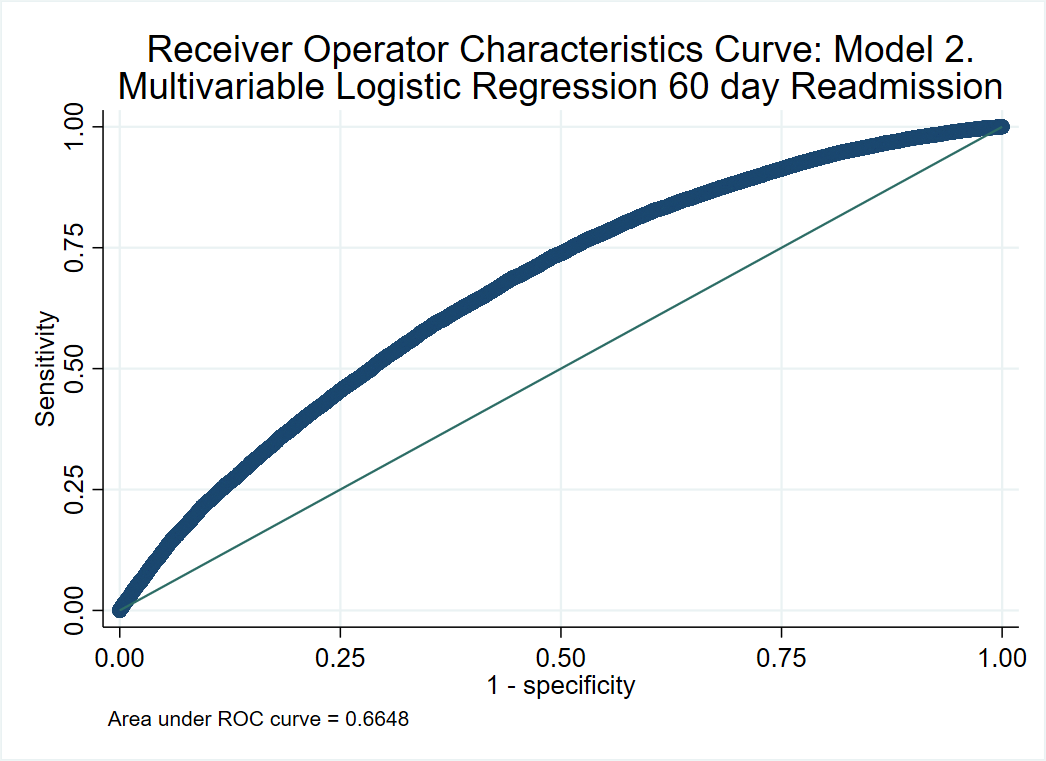


Supplemental Figure 4.3


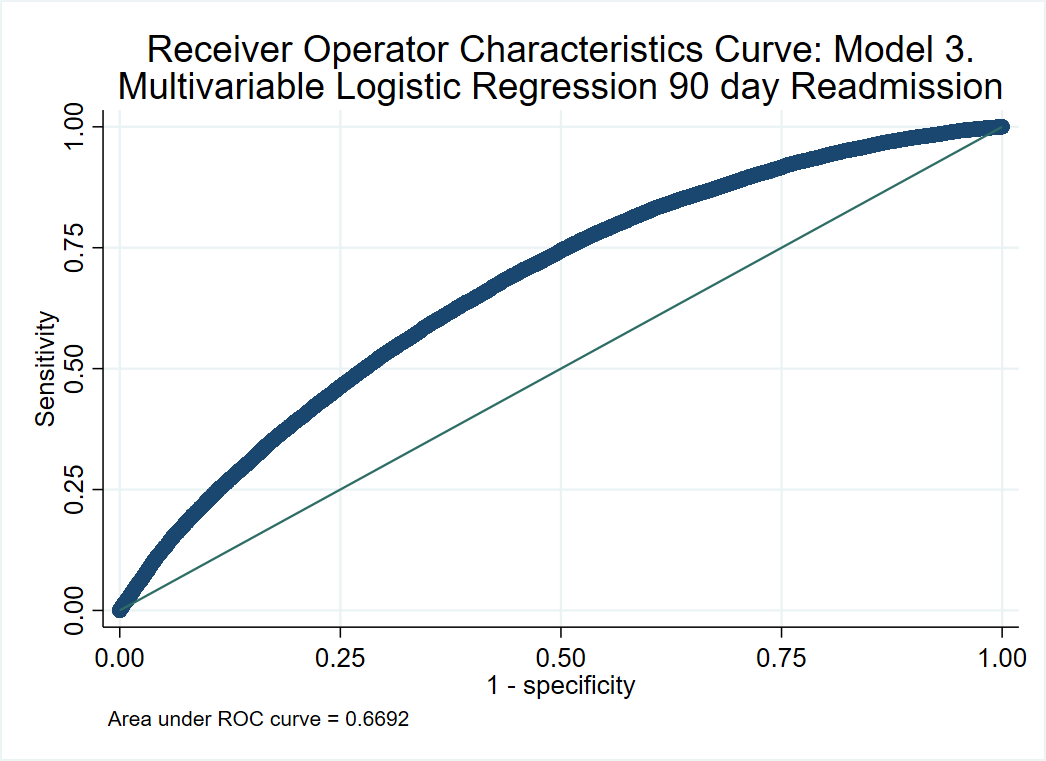

Supplement: Supplementary file 4 [file Data_Sheet_4.docx]
